# The Vulnerability of People to Landslides: A Case Study on the Relationship between the Casualties and Volume of Landslides in China

**DOI:** 10.3390/ijerph14020212

**Published:** 2017-02-21

**Authors:** Qigen Lin, Ying Wang, Tianxue Liu, Yingqi Zhu, Qi Sui

**Affiliations:** 1 Key Laboratory of Environmental Change and Natural Disaster of Ministry of Education, Beijing Normal University, Beijing 100875, China; linqigen@mail.bnu.edu.cn (Q.L.); liutianxue@mail.bnu.edu.cn (T.L.); Zhuyingqi@mail.bnu.edu.cn (Y.Z.); suiqi@mail.bnu.edu.cn (Q.S.); 2 Academy of Disaster Reduction and Emergency Management, Beijing Normal University, Beijing 100875, China

**Keywords:** landslide, vulnerability, casualties, volume, uncertainty, China

## Abstract

The lack of a detailed landslide inventory makes research on the vulnerability of people to landslides highly limited. In this paper, the authors collect information on the landslides that have caused casualties in China, and established the *Landslides Casualties Inventory of China*. 100 landslide cases from 2003 to 2012 were utilized to develop an empirical relationship between the volume of a landslide event and the casualties caused by the occurrence of the event. The error bars were used to describe the uncertainty of casualties resulting from landslides and to establish a threshold curve of casualties caused by landslides in China. The threshold curve was then applied to the landslide cases occurred in 2013 and 2014. The validation results show that the estimated casualties of the threshold curve were in good agreement with the real casualties with a small deviation. Therefore, the threshold curve can be used for estimating potential casualties and landslide vulnerability, which is meaningful for emergency rescue operations after landslides occurred and for risk assessment research.

## 1. Introduction

Landslides cause severe casualties and property loss in many regions. With human activities continually expanding to mountainous areas and with the global environment change, the frequency of the occurrence of landslides shows an apparent growing tendency [1]. According to the definition of Varnes [2], landslide risk refers to the expected degree of loss due to a landslide. The risk is determined by the product of the landslide hazard and the vulnerability. Landslide hazard and vulnerability information are required for a landslide risk assessment. Landslide hazard is defined as the probability that a specified magnitude of landslide occurs within a period of time and within a given area [2,3]. The literature concerning landslide hazard assessment is extensive [4,5,6,7,8,9,10].

However, the research on the vulnerability to landslide is relatively rare. Vulnerability refers to the degree of loss to a given element at risk due to the occurrence of a specified magnitude of hazard event [2]. Specifically, vulnerability is the relationship between the damage degree of the elements at risk and the intensity of the disaster. However, studies examining vulnerability to landslides are limited. The estimation of landslide vulnerability is highly complex due to several reasons. Glade [11] pointed out that the vulnerability of different elements at risk is variable during the similar hazard processes, and the temporal probability of people being exposed to the landslide is also variable. Moreover, the ability of different groups to cope with disasters affect the vulnerability of populations, and landslide susceptibility varies. In addition, landslide vulnerability is also affected by factors including velocity, block mass, impact angle of the landslide, position of the wall impact point, detailed geometry of the wall, and strength of the material. It is very difficult to deal with all of these factors [12].

At present, there is no uniform methodology to quantitatively assess the vulnerability of various elements at risk to different types and magnitudes of landslides [13]. Therefore, in actual landslide risk assessment, the most common method is to set the landslide vulnerability of different elements at risk to a constant 1, that is, to believe that the elements at risk will be completely damaged and lost, or to assign the vulnerability on the basis of expert knowledge and experience [14]. Several scholars study the vulnerability of building, road, and land use to landslide by the methods of vulnerability curve, vulnerability matrix, and vulnerability indicators [15,16,17,18,19,20,21,22,23,24,25,26,27,28,29,30,31]. Catani et al. [16] surveyed the loss ratio of such land uses as grassland, pasture, forest, and fruit forest under different landslide volumes and established vulnerability curves for various land uses. Remondo et al. [17] established a landslide inventory for the Deva Valley area in Northern Spain, surveyed both the relevant loss caused by landslides and the replacement costs of elements at risk in detail, and established vulnerability indicators for buildings, roads, and land in this area.

Eidsvig et al. [19] built a semiquantitative indicator-based model considering the influencing factors about a community’s ability to prepare for, deal with, and recover from the damage and loss associated with landslides to assess the relative socioeconomic vulnerability to landslides of the communities in Europe. Martha and Kumar [18] obtained the damage situation of buildings through comparing shape, color, and texture in remote sensing images before and after a landslide and assessed the vulnerability of buildings, agricultural land, and roads. Galli and Guzzetti [20] assigned the damage proportion of buildings in Umbria, Italy on the basis of several criteria and established a vulnerability threshold curve between the landslide area and the damage proportion of buildings and roads. Fotopoulou and Pitilakis [23,24] adopted a dynamic non-linear finite difference slope model to estimate the differential permanent landslide displacements at the building’s foundation level. The obtained differential permanent displacements are imposed at the foundation level to assess the building’s response to differing permanent seismic ground displacements based a finite element code. Finally, the fragility curves were built to assess the vulnerability of low-rise reinforced concrete buildings subjected to earthquake-induced slow-moving earth slides. Mavrouli et al. [25] used the particle finite element method to model the impact of the rockfall (velocity and size) to the masonry structure building. Then the finite element method was adopted to evaluate the mechanical properties, model the masonry structure, and calculate the internal stresses. Finally, a failure criterion was applied to calculate the damage and vulnerability of masonry structures to rockfalls were assessed. Papathoma-Köhle et al. [27] used nonlinear regression approaches to fit the vulnerability curve between the intensity of debris flow and the percentage of building loss due to the impact of debris flow. The obtained vulnerability curve was applied in Gand and Ennewasser villages in Italy. Papathoma-Köhle et al. [26] also reviewed the existing methods for physical vulnerability assessment for mountain hazards and identified difficulties in their implementation and differences between them. Finally, six future needs in the field of vulnerability assessment were proposed including user-friendliness of the method, the selection of all the relevant indicators, the transferability of the method, etc. Quan Luna et al. [28] used the FLO-2D program to model debris flow depth, impact pressure, and kinematic viscosity. Then, physical damage in terms of the ratio between monetary loss and reconstruction value was quantified. Finally, three different empirical vulnerability curves were obtained, which are functions of debris flow depth, impact pressure, and kinematic viscosity, respectively. Silva and Pereira [29] used the semi-quantitative method to assess the physical vulnerability of buildings and the associated potential losses resulting from the occurrence of shallow slides at the regional scale in the North of Portugal. Totschnig and Fuchs [30,31] used torrent events in the Austrian Alps to establish the quantitative vulnerability curve of the residential buildings to torrent. The results show a modified Weibull distribution and a modified Frechet distribution to fit best to the observed damage pattern in terms of absolute and relative values, respectively. Studies on examining the vulnerability of people to landslides are even more limited. Finlay [32] provided several recommended ranges and values of the vulnerability of a person in an open space, in a vehicle, or in a building derived from historical records of Hong Kong based on opinions of the expert. Murillo-García et al. [33] used the Spatial Approach to Vulnerability Assessment model to consider the indicators of Exposure, Sensitivity, and Lack of Resilience and assessed the vulnerability of population to landslide by multiplying them in every spatial unit in Pahuatlán, Mexico. Ferlisi et al. [34] assessed rockfall risk to persons traveling in vehicles along the Amalfi coastal road in Italy. The Rockfall Hazard Rating System (RHRS) method was used to qualitatively evaluate rockfall risk in different sections along the road considering the factors such as slope height, ditch effectiveness, and average vehicle risk. Then, the rockfall risk to persons traveling in vehicles was calculated under three hypothesized scenarios based on the quantitative risk assessment (QRA) procedures. The estimated societal risk is too high and cannot be tolerated. However, such models are based on the theory of disaster to build vulnerability models and has been not been subjected to enough practical testing.

Generally speaking, the non-uniform landslide intensity characteristic index and the lack of detailed historical landslide inventories have led to few studies investigating landslide vulnerability. Therefore, in this paper, the authors attempt to establish an empirical relationship between landslide intensity and the casualties caused by the occurrence of the event to fill in the gap in such studies. The authors collect and build a *Landslide Casualties Inventory of China* through data collection on the basis of multiple channels, and utilize the landslide cases between 2003 and 2012 as training samples to develop an empirical relationship between the volume of a landslide and the casualties caused by the occurrence of the event, that is, the casualties threshold curve, and apply this casualties threshold curve to data from 2013 to 2014 for verification. The casualties threshold curve proposed in this paper can be used to estimate possible casualties caused by a landslide quickly and provide effective information to emergency rescue.

## 2. Data and Methods

### 2.1. Landslide Casualties Inventory of China

**Figure 1:**
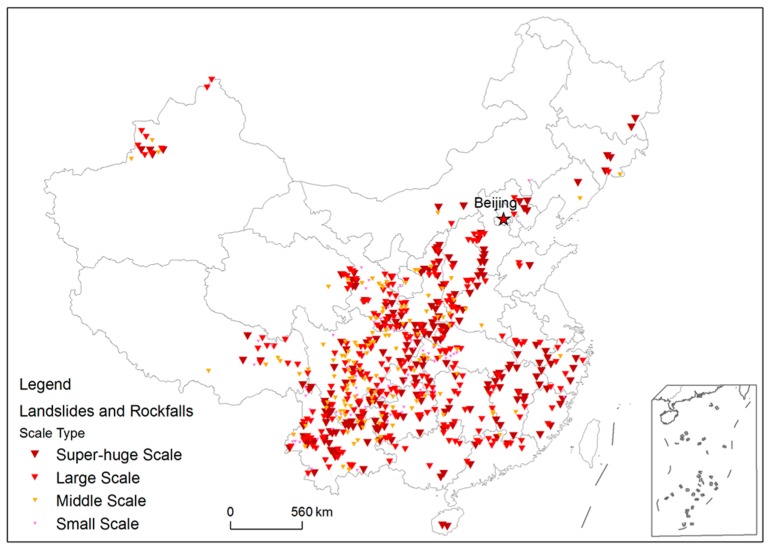
Landslide and rockfall distribution map in China.

**Figure 2:**
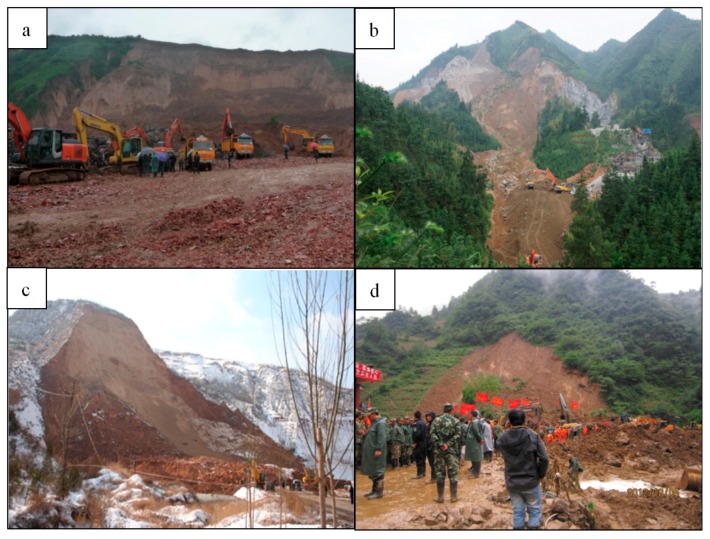
Cases of landslide: (**a**) Landslide in Baqiao District, Shaanxi on 17 September 2011; (**b**) Landslide in Quanzhou County, Guangxi on 9 May 2011; (**c**) Rockfall in Zhongyang County, Shanxi on 16 November 2009; (**d**) Landslide in Yiliang County, Yunnan on 4 October 2012.

China is one of the countries with the most severe geological disasters in the world. According to the statistics of China Statistic Yearbook, between 2000 and 2013, 2.5 × 10^4^ geological hazards occurred every year, which resulted in the deaths of 744 persons, injured 2564 persons. Landslides and rockfalls occurred the most frequently, accounting for 90.1% of all geological hazards [35]. Figure 1 shows the distribution of landslide and rock fall disaster map of China compiled by the China Institute for Geo-Environment Monitoring [36]. Landslides and rockfalls in China are mainly distributed in the Western Sichuan Mountainous Area and the Yunnan-Guizhou Plateau Region, the Southeast Hilly Area, North of the Loess Hills, and the Qinba Mountains Area and the Northwest Plateau Region. In recent years, landslides and rockfalls occurred frequently in these areas resulting in severe casualties. Figure 2 shows four cases of landslides in Guangxi, Yunnan, and Shanxi Province. Therefore, it is urgent to have landslide risk assessments for the identification and management of potential high landslide risk areas.

In the present study, the term landslide refers to the wider definition of the landslide with the exception of debris flow. The reason we excluded debris flow is that the amount of casualties caused by debris flow in China is more seriously than that of other landslides. For instance, the Zhouqu debris flow occurred on 8 August 2010 in Gansu Province, northwestern of China resulted in 1765 deaths. On 10 June 2005 in Heilongjiang Province, northeast China, the shalan debris flow caused 117 deaths.

The inventory of landslides in China has collected landslide information from four different data sources. The first source is the National Geological Hazard Bulletin [37], prepared by the Geological Environment Monitoring Institute of China. The Bulletin primarily includes an overall introduction to the frequency of occurrence of geological hazards in China and the number of losses in the year, a distribution and comparison with the past, and detailed survey information of serious geological hazard. The available time range for acquiring the data of the National Geological Disasters Bulletin is between 2004 and 2014, and the annual bulletin can be downloaded from the website (http://www.cigem.gov.cn/). The Geological Environment Monitoring Institute of China conducts relevant survey and research, such as geological hazard monitoring, forecasting, and early warning. The institute’s data quality is reliable; in particular, the field investigations of serious geological hazard events are accurate and credible.

The second data source is the Yesterday Disaster Report [38], prepared by the National Disaster Reduction Center of China. Since 2004, reports on disasters that have occurred in China (including earthquake, geological disaster, flood, drought, typhoon, and lightning strike) are summarized and compiled every day. The Ministry of Civil Affairs is responsible for natural disaster rescue in China; therefore, the Yesterday Disaster Report primarily investigates casualties and property loss caused by disasters and examines loss caused by disasters in detail. Landslide information that caused casualties in China is screened and obtained out of the Yesterday Disaster Report.

The third data source is the Geological Disaster/Hazard Report [39]. Since July 2011, the Ministry of Land and Resources issued an introduction on its website (http://www.mlr.gov.cn/dzhj/dzzh/zqxqbg/index.htm) to the geological disaster/hazard that have occurred all over China every day. The fourth data source is any news report on landslides in China, acquired through searching “geological hazard”, “geological disaster”, “landslide”, “casualties”, and other keywords on Google, to obtain rockfall and landslide information in news reports.

Finally, the authors summarize landslides that caused casualties from 2003 to 2014, collected and arranged data out of the four different data sources including the National Disaster Reduction Center, the Ministry of Civil Affairs, the Ministry of Land and Resources, the China Geological Environmental Information Website, and news searches. Landslide events that caused casualties were proofread according to the time and location of occurrence. Repeated disasters were eliminated. These landslide cases were uniformly arranged by such factors as type, occurrence time, location, intensity, casualties, and loss. At last, the Landslide Casualties Inventory of China were established including 602 landslide casualty events, 4157 casualties, and 2405 deaths. Details of any specific data source are referred in Table 1.

Based on the Landslide Casualties Inventory of China, the authors select landslide event with detailed magnitude information which can be used to develop an empirical relationship between landslide magnitude and casualties caused by the occurrence of the event. One hundred and fifty-six landslide events include detailed volume and casualty information. The authors use 100 landslide cases occurred between 2003 and 2012 to establish a landslide casualties threshold curve and apply the casualties threshold curve to the remaining 56 landslide cases between 2013 and 2014 for validation.

### 2.2. Methods

In this study, we used the average volume and causalities in different landslide groups to analyze and develop an empirical relationship between the volume of landslide and casualties caused by the occurrence of the event. Detailed steps are described as follows.

Among 100 landslide casualties events that occurred between 2003 and 2012 used for building the empirical curve, a landslide with the volume of 1 m^3^ in Qidaohe, Xintian Town, Wanzhou District, Chongqing—due to continuous heavy rain on 21 June 2007—had the smallest volume; while a large-scale mountain landslide in Jiwei Mountain, Tiekuang, Wulong County, Chongqing on 5 June 2009 and a landslide in Baofeng Village, Guanmen, Nanjiang County, Bazhong, Sichuan on 18 September 2011 had the largest volume, 5 × 10^6^ m^3^. The volumes in different landslide cases are notably different. It is unreasonable and inadvisable to analyze them all together. To solve this problem, the method of analyzing the average vulnerability suggested in the literature [12] was adopted. Therefore, according to criteria of similar volume within a group, and large volume differences between groups, the authors classified 100 landslide cases into 16 groups in accordance to their volume. Then, the average casualties caused by a landslide were calculated for each group. The representative volume and the average causalities in each landslide group can be analyzed. A detailed classification threshold, average volume, and volume standard deviation can be seen in Table 2.

Although most of the causes of casualties can be explained by the volume of landslides, there still exist some uncertainties due to the influence of other factors such as the velocity of the landslide, the location of the people (outdoors, in the building, in a vehicle), the population density and so on. To describe the uncertainties of casualties caused by landslides, the authors use an error bar and its standard error. The formula is as follows:

(1) $$SE=\frac{S}{\sqrt{n}}=\sqrt{\frac{\sum_{1}^{n}{\left(x_{i}-\bar{x}\right)}^{2}}{n\times \left(n-1\right)}}$$

where *SE* is standard error, S is sample standard deviation, x is sample value, $\bar{x}$ is sample average value, and n is sample number.

The standard error of casualties caused by landslides in groups of different volumes was calculated according to the Formula (1). The upper limit and lower limit threshold value of casualties caused by the occurrence of the landslide event were obtained by plus/minus one standard error on the basis of the average value. An error bar was adopted to indicate the upper limit and lower limit threshold value of casualties, that is the uncertainty.

The final step is to fit the upper limit or lower limit threshold value curve of casualties caused by a landslide. In this study, a linear function, exponential function, logarithmic function, high order polynomial, and power function were used to fit the threshold curve. The R Square was applied to assess the fitting results.

## 3. Results and Validation

**Figure 3:**
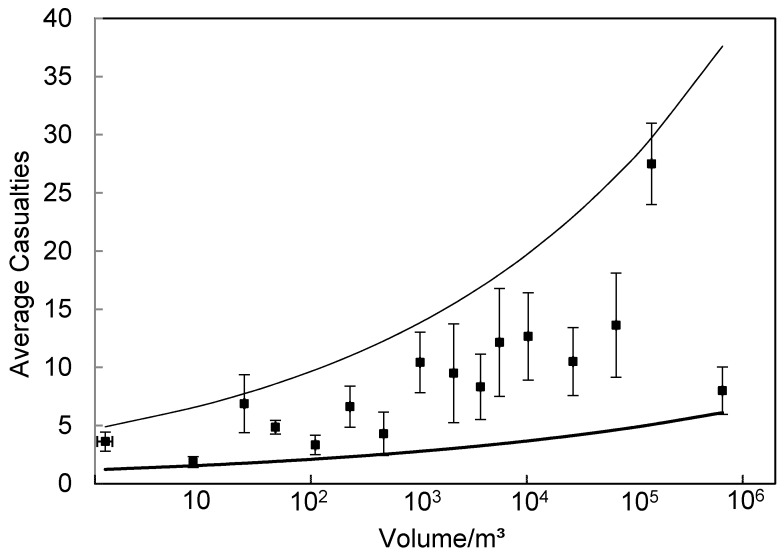
Empirical relationship between volume of landslide and the casualties caused by the occurrence of the event (the black dot in the figure indicates average casualties, the error bar is standard error, indicating the uncertainty of casualties caused by landslide; thin line/thick line respectively indicate the maximum and minimum threshold values of the landslide casualties curve).

For average casualties caused by a landslide in groups of different volumes, see Figure 3. The horizontal axis shows the logarithmic volume of a landslide, while the longitudinal axis shows the average casualties caused by that landslide. The figure shows that the casualties caused by the occurrence of the landslide are obviously related to the volume of the landslides. With the gradual increase of the volume of a landslide, the casualties caused by the occurrence of the event also increase constantly. For example, when the volume of a landslide is less than 1000 m^3^, the average casualties are within 10, when the volume of the landslide is 1000 m^3^–500 × 10^3^ m^3^, the casualties are approximately 10–20, and when the volume of a landslide is more than 500 × 10^3^ m^3^, more casualties are caused. There are still casualties caused when there is nearly 0 volume of landslide in Figure 3. It is because these landslides occurred along the road or the scenic areas which increased the probability of casualties. The size of the error bar also shows that when the volume of a landslide is small, the uncertainty of casualties caused is small. With the increase of the volume of a landslide, the uncertainty exhibits a growing tendency.

The fitting results of different functions are shown in Table 3. For the maximum threshold curve, the power function has the highest R-square with the value of 0.973. For the minimum threshold curve, the logarithmic function has the highest R-square with the value of 0.974 following by the power function with an R-square of 0.964. Based on the fitting performance of the maximum/minimum threshold curves, the power function was finally selected to represent the threshold value curve of casualties caused by landslides. In addition, it has been shown in the literature that the power function is suitable for the fitting of the landslide vulnerability curve. Galli and Guzzetti [20] adopted the power function to fit the landslide vulnerability curve because the power function is simple and performs reasonably well. Furthermore, the power function suggests a self-similar scaling behavior of the landslide damage which allows the damage information for small landslides to be applied for estimation of the vulnerability to large landslides and vice versa. The fitting result of the power function, that is the maximum and minimum landslide casualties threshold curves, are shown in Table 4.

The landslide casualties threshold curve that was established based the landslide cases occurred between 2003 and 2012 was applied to 56 landslide cases which were occurred between 2013 and 2014 to verify the results of the landslide casualties threshold value curve. In 56 landslide cases from 2013 to 2014 with detailed intensity and casualties, a rockfall in Huangchang Village, Mawu Town, Shizhu Tujia Autonomous County, Chongqing on 10 September 2013 had the smallest rockfall volume—2 m³—while a landslide in Changli County, Qinhuangdao, Hebei on 16 November 2013 had the largest rockfall and landslide volume—4 × 10^6^ m³. The volume of the landslides are within 1–5 × 10^6^ m³ and can be used to validate the landslide casualties threshold curve.

**Figure 4:**
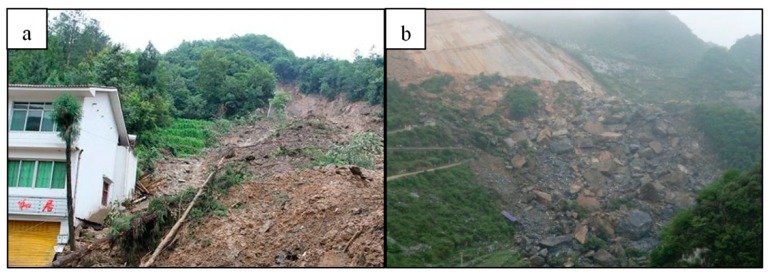
(**a**) Landslide in Qingchuan County, Sichuan; (**b**) Landslide in Nayong County, Guizhou.

Table 5 shows the information of landslide casualty events with a volume of more than 1000 m³ among the landslide cases from 2013 to 2014, and the actual casualties are compared with the casualties estimated through the landslide casualties threshold curve. In 21 of 26 landslide events, casualties are correctly estimated with an accuracy rate of more than 80%. In the landslide events which the casualties are wrongly estimated by the landslide casualties threshold value curve, the estimate deviation is used to assess the estimated error. Table 5 shows that the deviation of the wrong estimation is small. Except for landslide events No 17, 21, 24–26 the casualties estimate deviations in the rest of the landslide events are correct. For event 24—the largest error—a landslide in Qingchuan County, Sichuan on 12 July 2013 (Figure 4a) was 20 × 10^4^ m³, 19 houses were damaged, but only one casualty was caused. When the landslide occurred at 16:10 p.m., adults were working while children were studying at school; therefore, casualties were few. In event 17, a 150 × 10^4^ m³ landslide (Figure 4b) in Shengli Village, Gukai, Nayong County, Guizhou on 16 May 2013 was caused by the demolition of an aggregate plant. There were three casualties, including two demolition employees of the NaYong Demolition Company and an employee of the plant, while villagers nearby had been transferred to a safe area, which prevented serious casualties. In events 21, 25 and 26, the error is more than 50%. The actual casualties in the events are 1–2, and it is difficult to forecast such a small casualty event precisely. However, for a small casualty event, a 50% forecasting error will not have a strong influence on the level of emergency rescue measures.

Therefore, the results of Table 5 show that the landslide casualties threshold curve established in this paper is good and can be applied in estimating casualties caused by landslides.

## 4. Discussion

By compiling a Landslide Casualties Inventory of China, the authors attempt to develop empirical dependencies between the volume of landslide and casualties caused by the occurrence of the event. From the average status of casualties caused by a landslide, when the volume of a landslide is less than 1000 m^3^, the average casualties are within 10; when the volume of a landslide is 1000 m^3^–500 × 10^3^ m^3^, the casualties are approximately 10–20; and when the volume of a landslide is more than 500 × 10^3^ m^3^, more casualties are caused. In addition, due to complexity of factors that affect casualties caused by a landslide, the specific casualties caused by a landslide with a certain volume will exhibit fluctuations around the average value. The size of the error bar in Figure 3 shows such uncertainty. The fluctuation of the error bar indicates that the increase in the volume of a landslide results in increasing uncertainty of casualties. The validation results suggested that the proposed method considering the average status and uncertainty for establishing an empirical relationship between landslide intensity and the loss caused by that landslide can be further applied to other geological hazards or a smaller scale with a complete landslide intensity and loss inventory.

However, casualties caused by landslides are affected by many factors, including velocity of landslide, specific type of landslide, location, time of occurrence and population density, the location of the people (outdoors, in the building, in a vehicle), plus any hazard defenses and resisting capacity (e.g., age and gender). Finlay [32] analyzed the influence of the location of the person (in an open space, in a vehicle, or in a building) on the vulnerability of a person based on experts’ knowledge. García et al. [33] used the indicators—including the total population index, population density index, population lower than 12-year-old index, female population index, etc.—to assess the vulnerability of population to landslide in every spatial unit in Mexico. Therefore, estimating casualties caused by landslides is a complex task. The landslide casualties threshold curve obtained in this paper from the development of an empirical statistical relationship between the volume of a landslide and the casualties caused by the occurrence of the event is a useful attempt, but there are still some limitations in actual application. For example, the landslide casualties threshold value curve is established on the basis of a landslide; therefore, it cannot be applied in assessing vulnerability to a debris flow and the casualties or loss caused by a debris flow. The indicator of landslide intensity also only considers the volume of a landslide; because the maximum landslide volume in Landslide Casualties Inventory of China is only 5 × 10^6^ m³, it cannot be applied to assessing landslides with a larger volume.

Two factors lead to limitation above: one is the incomplete landslide inventory. The scattered occurrence of landslides makes it difficult to collect data and arrange a large number of complete landslide casualties and loss information. Therefore, in this paper, the authors can only utilize limited landslide cases with detailed information, specifically 100 landslide events occurred between 2003 and 2012 to establish the maximum/minimum casualties threshold curve and 56 landslide events between 2013 and 2014 were used to verify the landslide casualties threshold value curve. The casualties caused by the occurrence of landslides are affected by many factors, which means that a landslide with the same volume may result in completely different casualty situations. Only a part of the casualties caused by a landslide can be explained through the volume of the landslide; there is a certain error that is reflected by the error bar results in Figure 3. Therefore, if the landslide casualties threshold curve can be established on the basis of a complete landslide database, a more stable and reliable result will be obtained, which should be determined and discussed in future studies.

## 5. Conclusions

In this paper, the authors collected information regarding casualties caused by the occurrence of the landslides in China from 2003 to 2014 and established the Landslide Casualties Inventory of China through multiple information sources. On the basis of landslide events that occurred between 2003 and 2012, the authors developed an empirical relationship between volume of landslides and casualties caused by the occurrence of the event. Utilizing an error bar to describe the uncertainty of casualties caused by a landslide, a landslide casualties threshold value curve is established. The authors verify the curve by using landslide events that occurred between 2013 and 2014, and the results indicated that this threshold value curve has good agreement with estimated casualties.

The landslide casualties threshold value curve can be used to estimate possible casualties caused by a landslide quickly and provide effective information to emergency rescue. Conversely, this curve can also be used to identify the vulnerability of people to landslides to a certain extent and can provide information for research on a quantitative landslide risk assessment study by combining the curve with landslide hazard assessment models.

## Figures and Tables

**Table 1:** Data source of landslide casualties database of China.

Data Source	Prepared By	Records	Approach
National Geological Hazard Bulletin	GEMIC	61	Website of GEMIC
Web news search	News website	130	Online collection
Yesterday Disaster Report	NDRCC	180	NDRCC
Geological Disaster/Hazard Report	MLRC	231	Website of MLRC

**Table 2:** Result of landslide groups according to their volume.

Group	Number	Average Volume (m^3^)	Range (m^3^)	Standard Deviation
1	8	6.26	1–20	3.73
2	7	40.71	20–100	20.09
3	8	121.25	100–200	29.49
4	7	235.71	200–500	47.56
5	6	550.00	500–1×10^3^	122.47
6	8	1154.38	1 × 10^3^–2 × 10^3^	266.21
7	7	2362.86	2 × 10^3^–4 × 10^3^	533.22
8	7	5142.86	4 × 10^3^–8 × 10^3^	852.17
9	6	10,533.33	8 × 10^3^–15 × 10^3^	1818.42
10	6	18,666.67	15 × 10^3^–25,000	2160.25
11	7	27,942.86	25 × 10^3^–45,000	2573.49
12	3	51,666.67	45×10^3^–100 × 10^3^	6506.41
13	6	134,166.67	100× 10^3^–200 × 10^3^	37,738.13
14	8	336,250.00	200 ×10^3^–600 × 10^3^	100,418.77
15	2	715,000.00	600 × 10^3^–1 × 10^6^	162,634.56
16	4	3,250,000.00	1 × 10^6^–5 × 10^6^	2,061,552.81

**Table 3:** Model fitting summary.

Equation	Maximum		Minimum	
	R-Square	Sig.	R-Square	Sig.
Linear	0.819	0.032	0.938	0.032
Logarithmic	0.890	0.013	0.974	0.013
Polynomial	0.938	0.032	0.938	0.032
Power	0.973	0.018	0.964	0.018
Exponential	0.543	0.103	0.805	0.103

**Table 4:** Maximum/Minimum threshold value curve of casualties caused by landslide.

	Min	Max	Landslide Volume Range
Casualties	$V_{vul}=0.98\times V_{vol}^{0.122}$	$V_{vul}=3.681\times V_{vol}^{0.155}$	$1<V_{vol}\leq 5\times 10^{6}$ m^3^
R-Square	0.964	0.973	
Sig.	0.018	0.018	

**Table 5:** Validation of the threshold value curve of landslide casualties.

No.	Time	Location	Volume/m³	Actual Casualties (Estimate Range)	Deviation ^1^/%
1	2014/8/24	Wangmo County, Guizhou	180,000	12 (4–24)	0
2	2013/7/27	Yongshan County, Yunnan	120,000	12 (4–23)	0
3	2014/10/28	Dongchuan District, Yunnan	100,000	12 (4–22)	0
4	2014/9/1	Yunyang County, Chongqing	20,000	12 (3–17)	0
5	2014/7/16	Anhua County, Hunan	16,000	11 (3–17)	0
6	2013/7/9	An County, Sichuan	1,000,000	10 (5–31)	0
7	2014/7/17	Zhijin County, Guizhou	14,000	8 (3–16)	0
8	2014/4/6	Ji County, Shanxi	2000	8 (2–12)	0
9	2013/8/20	Hezhang County, Guizhou	2000	7 (2–12)	0
10	2013/11/16	Changli County, Hebei	4,000,000	6 (6–39)	0
11	2014/8/6	Ludian County, Yunnan	3,000,000	6 (6–37)	0
12	2014/7/4	Xinhuang County, Hunan	105,000	6 (4–22)	0
13	2013/2/18	Kaili, Guizhou	100,000	5 (4–22)	0
14	2014/9/23	Xingyi, Guizhou	2000	5 (2–12)	0
15	2013/8/19	Guiping, Guangxi	1000	5 (2–11)	0
16	2014/6/28	Daguan County, Yunnan	60,000	4 (4–20)	0
17	2013/5/16	Nayong County, Guizhou	1,500,000	3 (6–35)	85
18	2013/9/4	Fushan County, Shanxi	30,000	3 (3–18)	0
19	2013/9/18	Gong County, Sichuan	30,000	3 (3–18)	0
20	2014/9/18	Yubei District, Chongqing	30,000	3 (3–18)	0
21	2014/7/21	Pan County, Guizhou	27,000	2 (3–18)	7
22	2013/4/1	Jinxiu County, Guangxi	1000	2 (2–11)	0
23	2013/7/9	Dujiangyan, Sichuan	1500	2 (2–11)	0
24	2013/7/12	Qingchuan County, Sichuan	200,000	1 (4–24)	334
25	2014/8/28	Hengshan County, Shaanxi	12,000	1 (3–16)	208
26	2013/6/18	Zhengan County, Guizhou	6000	1 (3–14)	183

^1^ Deviation = Min (estimate threshold value-actual value)/actual value).
